# Hypoxia-induced oxidative stress and mitochondrial damage initiate ferroptosis in *Cryptocaryon irritans*, a protozoan parasite of marine fish

**DOI:** 10.1007/s44154-025-00275-0

**Published:** 2026-01-12

**Authors:** Baotun Wang, Zhi Luo, Jingyu Zhuang, Zhicheng Li, Xueli Lai, Huicheng Wu, Qing Han, Jizhen Cao, Hebing Wang, Chuanfu Dong, Anxing Li

**Affiliations:** 1 State Key Laboratory of Biocontrol/Guangdong Provincial Key Laboratory of Improved Variety Reproduction in Aquatic Economic Animals and Institute of Aquatic Economic Animals, School of Life Sciences, Sun Yat-Sen University, Guangzhou, Guangdong Province 510275 PR China; 2 University Joint Laboratory of Guangdong Province, Hong Kong and Macao Region on Marine Bioresource Conservation and Exploitation, College of Marine Sciences, South China Agricultural University, Guangzhou, 510642 PR China

**Keywords:** *Cryptocaryon irritans*, Hypoxia, Metabolic reprogramming, Ferroptosis, Oxidative stress

## Abstract

*Cryptocaryon irritans* is an obligate parasitic ciliate that significantly endangers marine fish. Hypoxia suppresses the development and hatchability of *C. irritans* during the tomont stage, which often develops on the seafloor under hypoxic conditions. Despite this knowledge, the underlying adaptation mechanisms of tomonts remain poorly understood. We aimed to determine how hypoxia reprograms tomont metabolism and whether ferroptosis contributes to hypoxia-induced vulnerability. Herein, metabolomic profiling revealed 2,964 differential metabolites under hypoxia. Notably, there were significantly elevated glucose levels, suggesting enhanced glycolytic activity. Enzymatic and qRT-PCR analyses further confirmed hypoxia-induced metabolic reprogramming, including increased hexokinase and pyruvate kinase activities and upregulation of glycolysis-related genes. Hypoxia also induced surface depressions, disrupted cell walls, mitochondrial deformation, reduced mitochondrial membrane potential, disrupted energy homeostasis, and increased NAD⁺/NADH ratio fluctuations and lactate accumulation. To probe ferroptotic susceptibility under hypoxia, hypoxic tomonts were exposed to the ferroptosis inducer erastin, resulting in a hatchability of 13% and promoting reactive oxygen species (ROS) accumulation, lipid peroxidation, and mitochondrial damage. Fluorescence staining revealed strong PI and ROS signals in hypoxic tomonts exposed to the ferroptosis inducer erastin. Notably, mitochondrial dysfunction was accompanied by Ca^2^⁺ and Fe^2^⁺ accumulation. Ferroptosis-related genes were upregulated at 24 h post-hypoxia induction. In contrast, *gpx4* and mitochondrial electron transport chain components were downregulated at 48 h post-hypoxia induction. These findings demonstrate that hypoxia triggers glycolytic reprogramming and mitochondrial dysfunction in *C. irritans*, whereas erastin induces ferroptosis under hypoxic stress. This study provides new insights into protozoan hypoxia adaptation and highlights ferroptosis as a potential therapeutic target for controlling parasitic infections in marine aquaculture.

**Graphical Abstract:**

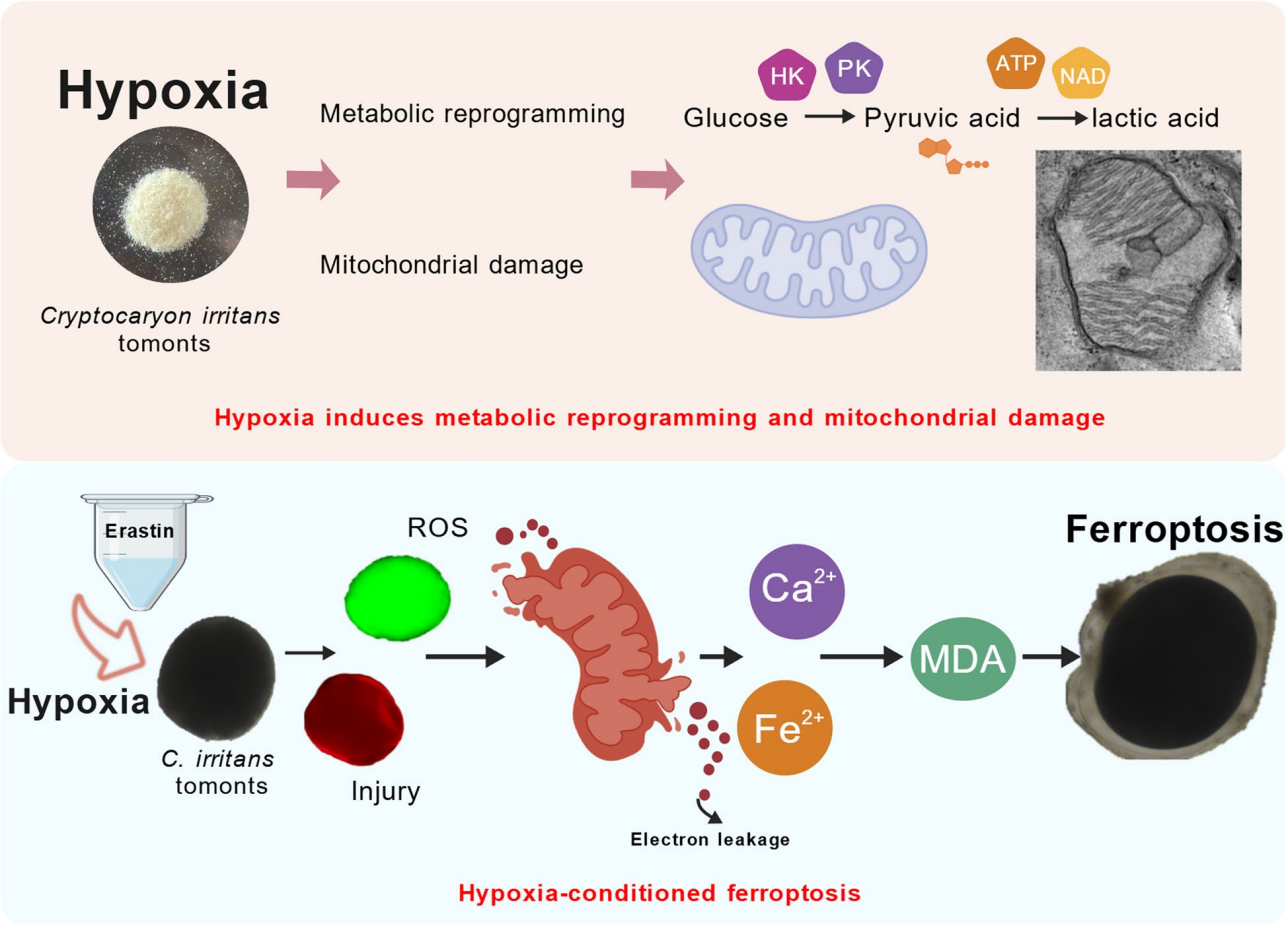

**Supplementary Information:**

The online version contains supplementary material available at 10.1007/s44154-025-00275-0.

## Introduction

*Cryptocaryon irritans * is an obligate parasitic ciliate found on marine teleost fishes. It follows a direct quadriphasic life cycle consisting of trophonts, protomonts, tomonts, and theronts (Li et al. 2021). Trophonts are the parasitic stage of *C. irritans*. Mature trophonts detach from the host, sink to the substrate, and subsequently transform into protomonts (Dan et al. 2006; Li et al. 2021). Protomonts are the motile stage of *C. irritans* that typically develop into tomonts within 2 h (Li et al. 2021). Tomonts are the most durable form of *C. irritans* during the extracellular phase, typically surviving for 3–4 days because of their immobility (Ma et al. 2017). *C. irritans* faces multiple environmental stressors in aquaculture, which alter their physiological functions and characteristics (Kong et al. 2022; Wang et al. 2025; Yin et al. 2023). Tomonts adhere to netting in the depths of seawater cages or to sediment on the seabed in marine environments, triggering cryptocaryoniasis outbreaks, despite being under hypoxic conditions (Jiang et al. 2022; Wang et al. 2025).

In higher metazoans, hypoxia-induced metabolic shifts are largely mediated by hypoxia-inducible factors (HIFs) (Loenarz et al. 2011). However, protozoa, including ciliates, adopt alternative adaptation mechanisms because of the lack of HIF homologs (Boxma et al. 2005; Graf et al. 2021; Lindmark and Müller 1973). Anaerobic ciliates, such as *Nyctotherus ovalis* and *Tritrichomonas foetus*, harbor mitochondria-derived organelles known as hydrogenosomes that support ATP synthesis under hypoxia (Boxma et al. 2005; Lindmark and Müller 1973). Hydrogenosomes, or their endosymbionts, possess genes for truncated electron transport chains and denitrification, which broaden the metabolic flexibility of protozoa survival under hypoxic conditions (Boxma et al. 2005; Graf et al. 2021; Hjort et al. 2010).

Hypoxia perturbs iron homeostasis and redox balance, increases reactive oxygen species (ROS), and promotes lipid peroxidation—biochemical changes that mechanistically intersect with ferroptosis (Liu et al. 2024; Zheng et al. 2023). Ferroptosis frequently occurs under hypoxic conditions in which disruption of the TCA cycle and glutaminolysis potentially contribute to ferroptotic vulnerability (Fuhrmann and Brüne 2017; Gan 2021; Gao et al. 2015; Murphy 2008). Ferroptosis is a form of regulated cell death characterized by iron-dependent lipid peroxidation and accumulation of ROS (Gan 2021). Notably, ferroptosis is driven by mitochondrial metabolism and redox imbalance (Gan 2021; Gaschler and Stockwell 2017; Liu et al. 2024). Ferroptosis inducers, such as Erastin and RSL3, suppress proliferation and enhance ROS toxicity in parasitic protozoa (Huang et al. 2023; Li et al. 2022; Saraiva et al. 2022). However, ferroptosis remains unexplored in *C. irritans*. Notably, mitochondria play a central role in executing ferroptosis because they are the major site of ROS generation and iron homeostasis.

Studies on the cell death of *C. irritans* have largely focused on apoptosis and cuproptosis induced by drugs or metals (Zahid et al. 2024; Zhao et al. 2023; Zhong et al. 2023). Tomonts develop at the sediment–water interface of marine cages, where low dissolved oxygen (DO) is common, making hypoxia an ecologically relevant stressor for *C. irritans* (Wang et al. 2025). Accordingly, we hypothesize that hypoxia-derived stress perturbs the metabolic state of *C. irritans* tomonts and may, in turn, enhance their susceptibility to ferroptosis. To test this, we combined morphological, metabolomic, biochemical, and molecular approaches to examine tomonts under hypoxia with and without the ferroptosis inducer, erastin. Clarifying this link will improve understanding of how hypoxia shapes parasite survival and—because the encysted tomont is the most treatment-refractory stage—may inform stage-targeted control strategies in aquaculture.

## Results

### Hypoxia-induced mitochondrial morphological alterations in *C. irritans* tomonts

**Fig. 1:**
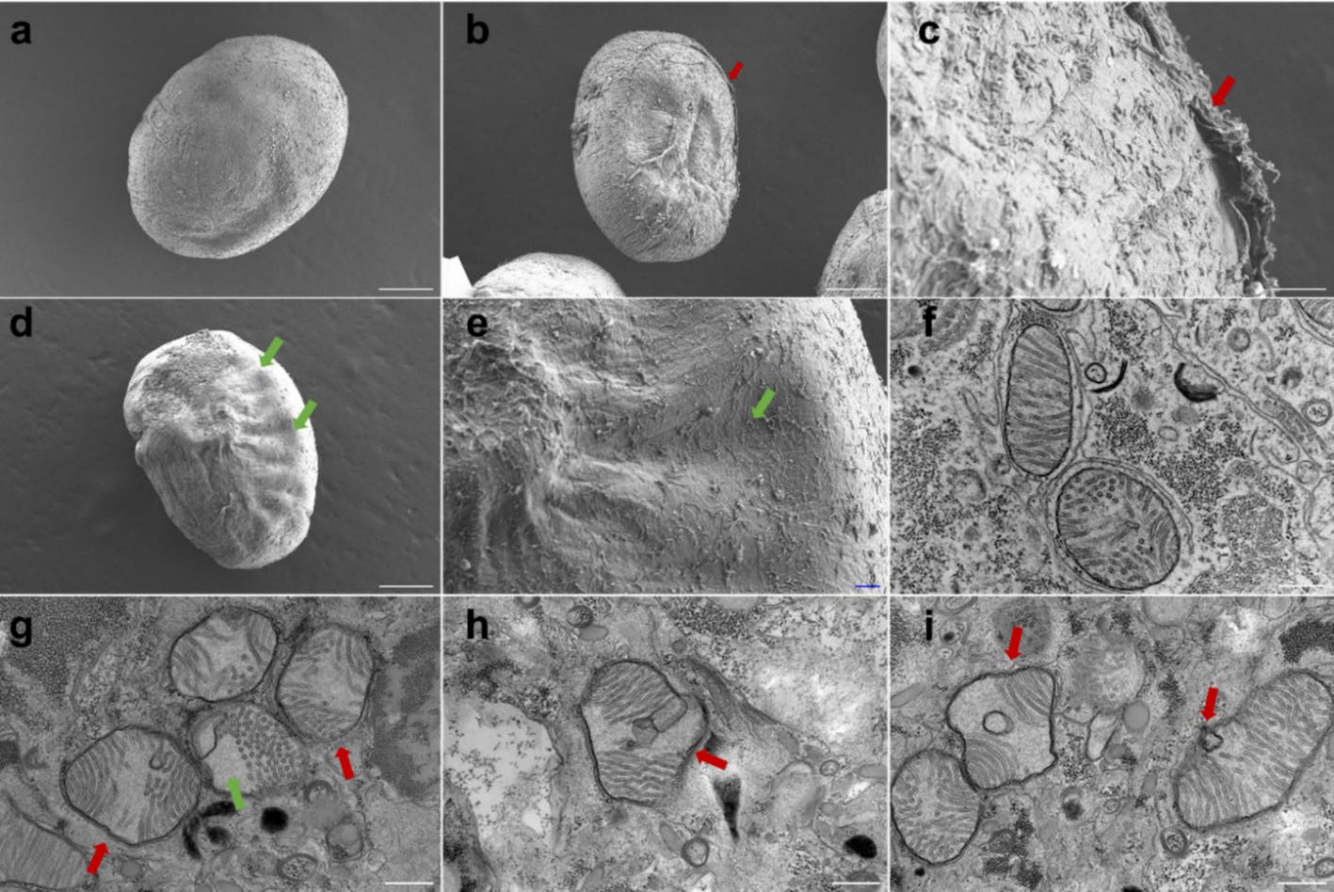
Ultrastructural alterations of *Cryptocaryon irritans* tomonts under normoxic and hypoxic conditions analyzed by scanning electron microscopy (SEM) and transmission electron microscopy (TEM). **a** SEM image of a tomont under normoxia, **b**–**e** SEM images of tomonts exposed to hypoxia. Red arrows indicate areas of cell wall rupture; green arrows highlight surface depressions. Panels (**c**) and (**e**) show magnified views of (**b**) and (**d**), respectively. Scale bars: 100 μm for (**a**), (**b**), and (**d**); 10 μm for (**c**) and (**e**). **f** TEM image of a tomont under normoxia. **g**–**i** TEM images of hypoxic tomonts. Red arrows denote mitochondria with disrupted cristae; green arrows indicate mitochondrial vacuolization. Scale bars: 500 nm for (**f**–**i**)

Because mitochondrial ultrastructure is a sensitive indicator of hypoxic stress in protozoa, we first examined whether moderate hypoxia elicits mitochondria-associated morphological changes in *C. irritans* tomonts. Tomonts displayed an oval shape with a smooth surface and well-preserved morphology (Fig. 1a), while their mitochondria exhibited clear double membranes and distinct tubular cristae under normoxic conditions (Fig. 1f). In contrast, tomonts exposed to hypoxia showed pronounced surface depressions, disrupted cell walls, and widened intercellular spaces (Fig. 1b-e). Mitochondria exhibited cristae loss and contained vacuole-like structures, suggesting substantial mitochondrial dysfunction during hypoxia (Fig. 1g-i).

### Hypoxia-induced metabolic changes in *C. irritans* tomonts

**Fig. 2:**
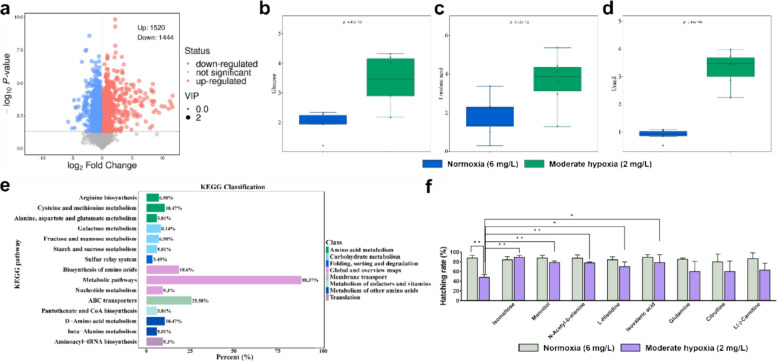
Metabolomic analysis of *Cryptocaryon irritans* tomonts under hypoxia. **a** Volcano plot shows the differential metabolites (*n* = 6). **b** Glucose, **c** Linoleic acid, and **d** Uracil content (*n* = 6). **e** KEGG pathway. **f** Effects of adding different exogenous metabolites on the hatching rate of *C. irritans* tomonts (*n* = 3). Statistical analysis in (**b**-**d** and **f**) was performed using a two-tailed Student’s t-test. Data are shown as mean ± SD. ‘n’ indicates biological replicates. *p < *0.05* and **p < *0.01*

Having established structural signs of hypoxic stress, we next profiled the metabolome under hypoxia to test whether hypoxia drives global metabolic reprogramming and impacts tomont viability. Metabolite profiles of *C. irritans* were significantly altered after 24 h of hypoxia exposure. OPLS-DA analysis revealed that t [1]P and t [1]O accounted for 23.8 and 13.9% of the variance, respectively, indicating good intra-group reproducibility and significant inter-group differences (Supplemental Figure S1). Hypoxia induced changes in 2,964 metabolites of *C. irritans*, with 1,520 upregulated and 1444 downregulated (Fig. 2a). DonutPlot analysis categorized the differential metabolites into lipids and lipid-like molecules (14.7%), organic acids and derivatives (12.35%), amino acids and peptides (4.83%), carbohydrates (2.35%), and other metabolites (65.77%) (Supplemental Figure S2). Additionally, KEGG enrichment analysis revealed that hypoxia primarily activated the metabolic pathway of *C. irritans* (88.37%) (Fig. 2e) and to some extent it increased the glucose content and enhanced the levels of linoleic acid and uracil (Fig. 2b-d). After 24 h of hypoxia, the malic acid content was significantly decreased (*P* < *0.01*), while the pyruvate content showed no significant change *(P* = *0.36*) (Supplemental Figure S3). Functionally, The hatching rates of tomonts in the absence of metabolites, in the normoxia (6 mg/L) and moderate hypoxia (2 mg/L) groups were 88.3 and 49%, respectively. Notably, the moderate hypoxia (2 mg/L) group exhibited a significantly lower hatching rate (*P* < *0.01*). However, the addition of isomaltose, mannitol, N-acetyl-β-alanine, L-histidine, isovaleric acid, glutamine, citrulline, and L(-)-carnitine in the normoxia (6 mg/L) group produced the hatching rates of tomonts that ranged from 80.1 to 89.7%, with insignificant differences among these hatching rates (*P* > *0.05*). Under hypoxia, the addition of isomaltose, mannitol, N-acetyl-β-alanine, L-histidine and isovaleric acid improved the hatching rates to 90.4, 79.3, 78.6, 70.8 and 79.3%, respectively, alleviating hypoxic damage. However, the addition of exogenous glutamine, citrulline, and L (-)-carnitine under moderate hypoxia (2 mg/L) group reduced the hatching rates to 60.7, 60.7, and 63.8%, respectively. Of note, the effects of the exogenous substances were not significantly different (*P* > *0.05*) (Fig. 2f).

### Hypoxia-induced changes in the energy metabolic profile of *C. irritans* tomonts

**Fig. 3:**
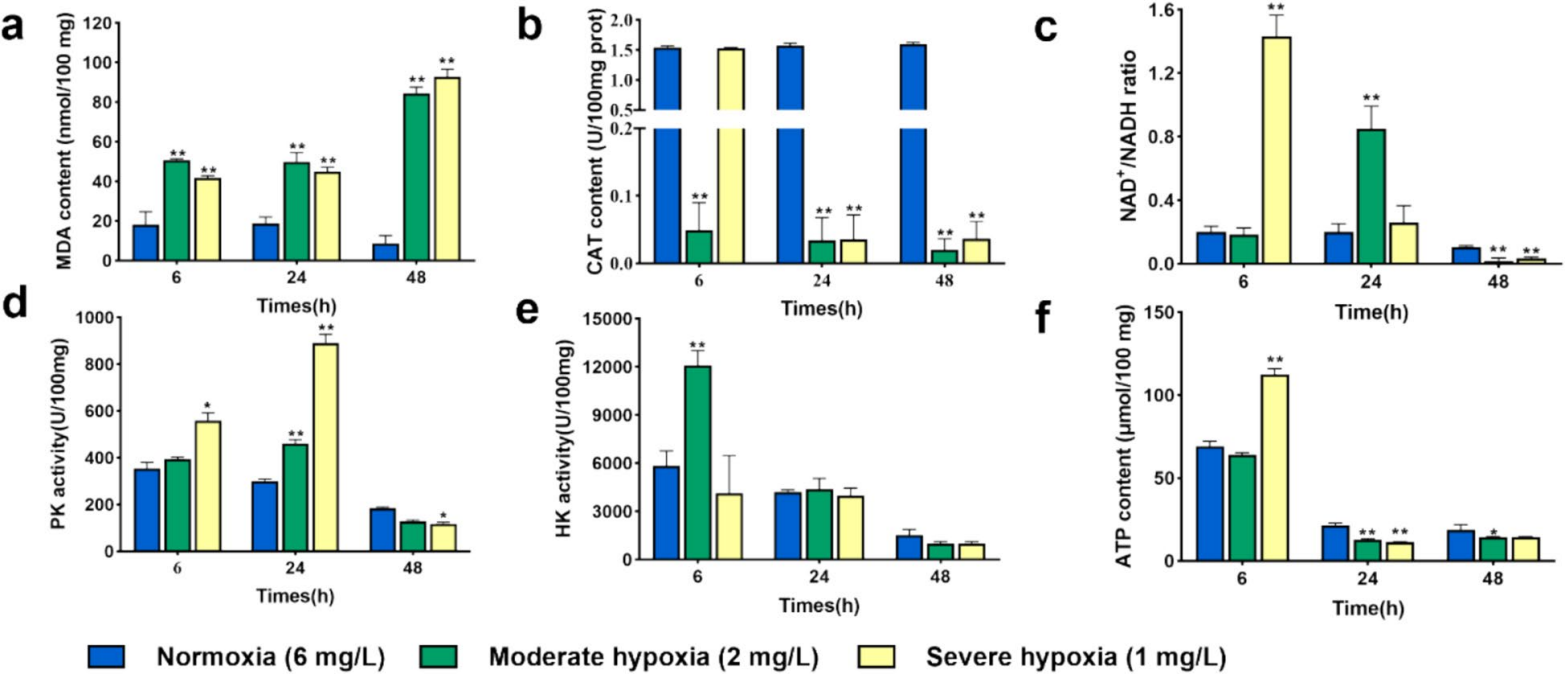
Metabolic enzyme activity analysis of *Cryptocaryon irritans* tomonts under hypoxia. **a** malondialdehyde (MDA), **b** catalase (CAT), **c** NAD^+^/NADH ratio, **d** pyruvate kinase (PK), **e** hexokinase (HK) and **f** ATP (*n* = 3). Activities of PK (at 6 and 48 h) and HK (at 24 and 48 h) were analyzed using the Kruskal–Wallis test followed by Dunn’s multiple-comparisons test. All other datasets were analyzed by one-way ANOVA with Dunnett’s multiple comparisons test. Data are presented as mean ± SD; n denotes biological replicates. * *p* < *0.05* and **p < *0.01*

To characterize hypoxia-driven effects on energy metabolism, we compared key energy- and redox-related readouts at early (6 h), mid (24 h), and late (48 h) phases. Compared with the normoxia group (6 mg/L), MDA content increased throughout under hypoxia (*P* < *0.01*), whereas CAT content declined from the mid phase onward (*P* < *0.01*) (Fig. 3a and b). In the moderate hypoxia (2 mg/L) group, the NAD⁺/NADH ratio increased in the mid phase, whereas in the severe hypoxia (1 mg/L) group it increased in the early phase (*P* < *0.01*) (Fig. 3c). Glycolytic activation appeared early and PK was elevated in both hypoxic groups at the mid phases, and HK increased in the moderate hypoxia (2 mg/L) group (*P* < *0.01*) (Fig. 3d and e). Energy supply showed a transient compensation followed by decline. ATP rose transiently in the early phase under severe hypoxia (1 mg/L), then decreased thereafter, while the moderate hypoxia (2 mg/L) group exhibited decreases from the mid to late phases(*P* < *0.01*) (Fig. 3f). Lactate levels increased in the late phase, with severe hypoxia (1 mg/L) group showing a significant elevation (*P* < *0.01*) (Supplemental Figure S4). Overall, hypoxia triggers early glycolytic activation with brief ATP compensation, then progresses to mid-late redox imbalance and lactate buildup, indicating worsening energetic stress.

### Hypoxic stress alters metabolic factor levels in *C. irritans* tomonts

**Fig. 4:**
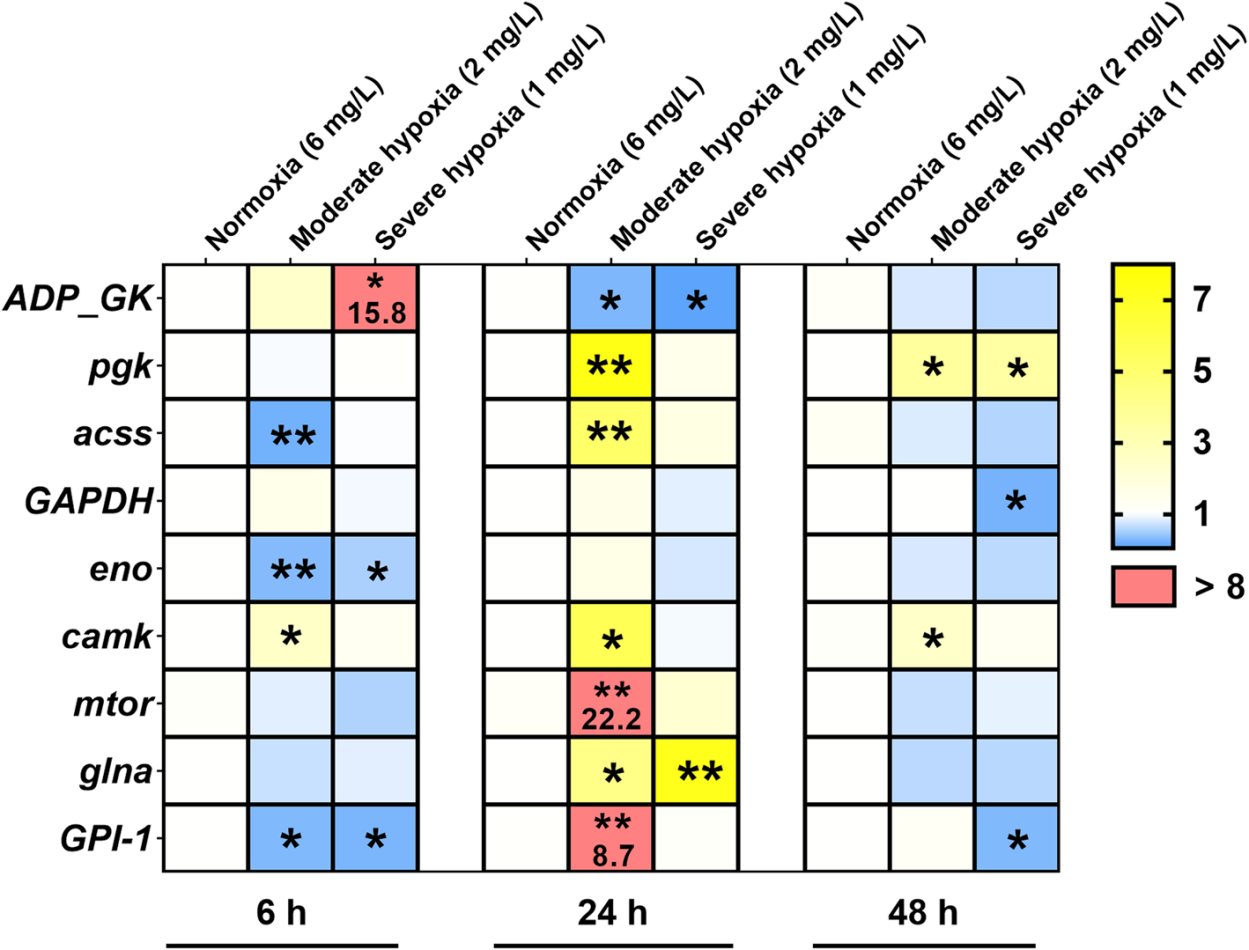
Heatmap displaying RT-qPCR expression profiles of metabolic genes in *Cryptocaryon irritans* tomonts. Yellow indicates gene upregulation, blue indicates downregulation, and red highlights genes with more than 8-fold upregulation. Statistical analysis was performed using one-way ANOVA with Dunnett’s multiple comparisons test. * *p* < *0.05* and **p < *0.01*

To keep a clear line of reasoning from hypoxia, via metabolic reprogramming, to ferroptotic pressure, we profiled transcripts governing glycolysis and energy/redox signaling across the early, mid, and late phases of hypoxia. In the early phase, *ADP_GK* and *camk* were induced, with *ADP_GK* increased 15.8-fold (*P* < *0.05*). By the mid phase, *pgk*, *acss*, *camk*, *mtor*, *glna*, and *GPI-1* were significantly up-regulated, with *mtor* and *GPI-1* rising 22.2-fold and 8.7-fold in moderate hypoxia (2 mg/L) group, respectively *(P* < *0.05*). In the late phase, *pgk* and *camk* remained significantly elevated (*P* < *0.05*). In contrast, the expression levels of *GAPDH* and *eno* were consistently downregulated throughout the hypoxia (Fig. 4 and Supplemental Figure S5). Collectively, the gene expression profile reflects a staged hypoxic response, with early activation, a mid-phase broad upregulation of glycolytic and signaling genes and late persistence of key drivers, while *GAPDH* and *eno* remain suppressed throughout, indicating coordinated metabolic rewiring.

### Erastin reduces the hatching rate of *C. irritans* tomonts under hypoxia

**Fig. 5:**
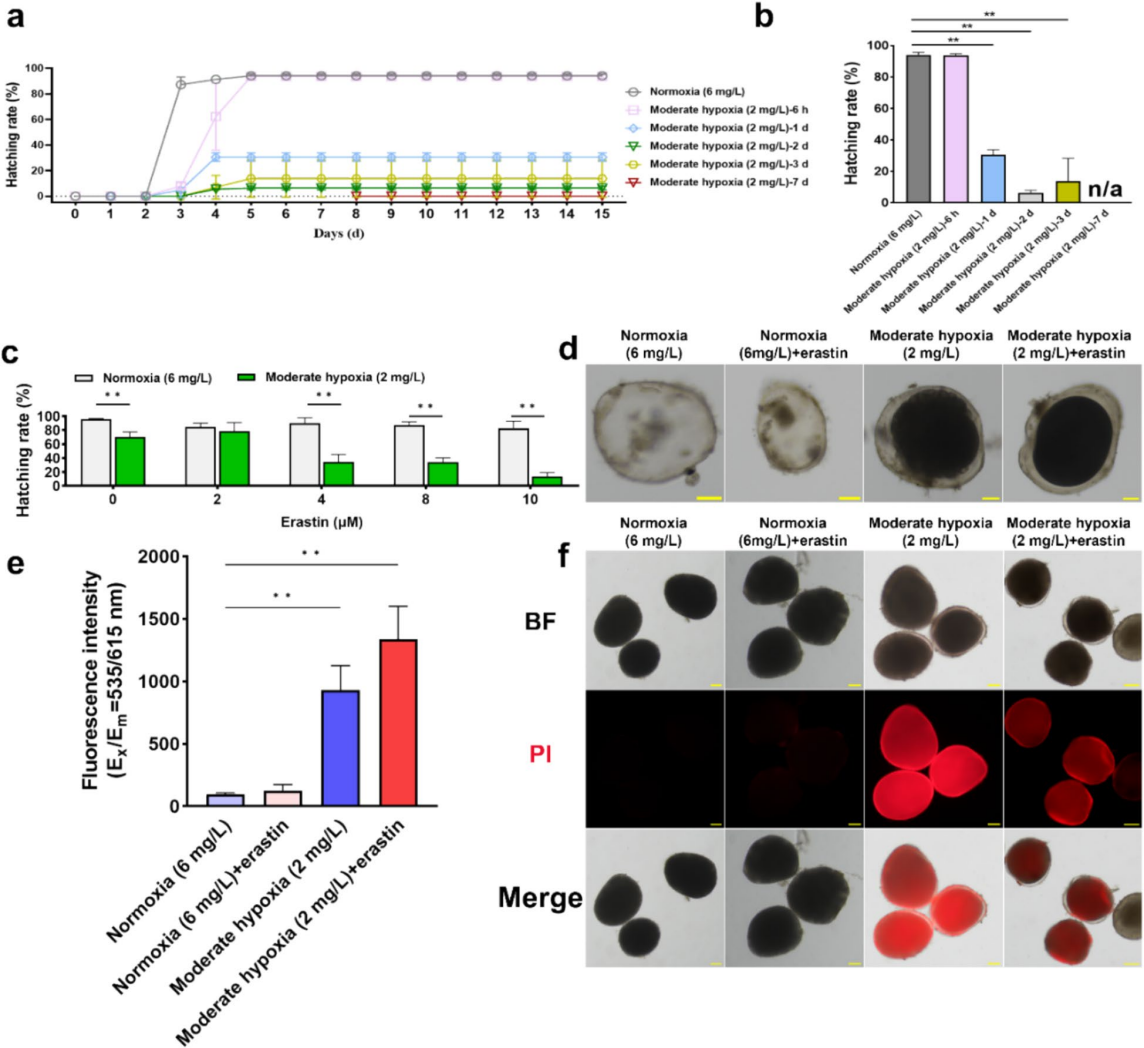
Inhibitory effects of erastin and hypoxia on *C. irritans* tomonts. **a** Hatching rates of *C. irritans* tomonts following exposure to hypoxia for different durations (6 h to 7 d), with subsequent reoxygenation (*n* = 3). **b** Hatching rates of *C. irritans* tomonts under different durations of hypoxia followed by 7 d of reoxygenation (*n* = 3). The moderate hypoxia (2 mg/L) group, which showed complete inhibition (0%), was included in the figure for reference but excluded from statistical analysis. (n/a) indicates not applicable. **c** Hatching rates of *C. irritans* tomonts treated with 2, 4, 8, and 10 μM erastin under normoxia (6 mg/L) and moderate hypoxia (2 mg/L) (*n* = 3). **d** Morphology of *C. irritans* tomonts exposed to hypoxia and erastin. Scale bars, 100 µm (two left panels) and 50 µm (two right panels). **e** Propidium iodide (PI) fluorescence intensity (E_x_/E_m_ = 535/615 nm) in tomonts under normoxia (6 mg/L), moderate hypoxia (2 mg/L), and erastin-treated (10 μM) (*n* = 3). **f** PI fluorescence staining images of *C. irritans* tomonts under E_x_/E_m_ = 535/615 nm at 48 h in each group. Scale bar, 100 µm. Statistical analysis in (**c**) was performed using a two-tailed Student’s t-test. Multiple comparisons in (**b**) and (**e**) were performed using one-way ANOVA with Dunnett’s multiple comparisons test. Data are shown as mean ± SD. ‘n’ indicates biological replicates. **p < *0.01*

To determine how hypoxia duration shapes developmental outcomes and whether reoxygenation restores normal hatching, we first quantified hatching after defined hypoxic exposures. After exposure to hypoxia for different durations, the *C. irritans* tomonts still hatched predominantly between days 3 and 4 post-treatment (Fig. 5a). On day 15, the hatching rate of the normoxia (6 mg/L) group was 94.2%. In contrast, the hatching rates of tomonts subjected to hypoxia for, 1, 2, and 3 d were 30.6, 6.5, and 13.9%, respectively (*P* < 0.01). Notably, tomonts exposed to hypoxia for 7 d failed to hatch after reoxygenation, with a hatching rate of 0% (Fig. 5b).

We next used erastin to test whether pharmacologic ferroptotic pressure further compromises the already fragile post-hypoxia hatching. Overall hatching was 96% under normoxia (6 mg/L) and 70.5% under moderate hypoxia (2 mg/L) group, indicating a highly significant hypoxic inhibition (*P* < *0.01*). Erastin did not affect the hatching rates of tomonts under normoxia but enhanced the inhibition of tomont hatching under hypoxia. The hatching rates of tomonts in the moderate hypoxia (2 mg/L) group were 34.6, 33.8, and 13.9%, with the addition of 4, 8, and 10 μM erastin, respectively (Fig. 5c). Tomonts in the normoxia (6 mg/L) and the normoxia (6 mg/L) + erastin groups successfully hatched theronts after the eighth day. In contrast, tomont death occurred in the moderate hypoxia (2 mg/L) and the moderate hypoxia (2 mg/L) + erastin groups. Tomont division phases were observed in the moderate hypoxia (2 mg/L) group, while only solid tomonts were observed in the moderate hypoxia (2 mg/L) + erastin group (Fig. 5d).

To test whether hypoxia damages tomont membranes and whether erastin intensifies this injury in a manner compatible with ferroptosis, we quantified PI uptake. PI staining revealed that the fluorescence intensities at Ex/Em = 535/615 nm in the normoxia (6 mg/L), normoxia (6 mg/L) + erastin, moderate hypoxia (2 mg/L), and moderate hypoxia (2 mg/L) + erastin groups were 95, 126.3, 929, and 1335.3, respectively (Fig. 5e). Hypoxia markedly increased PI fluorescence, which became strikingly intense when combined with erastin (Fig. 5f). Together, these results indicate hypoxia-induced membrane damage that is exacerbated by erastin, a pattern compatible with ferroptotic vulnerability; accordingly, we next interrogated ferroptosis-related markers to test this interpretation directly.

### Erastin induces oxidative stress in *C. irritans* tomonts under hypoxia

**Fig. 6:**
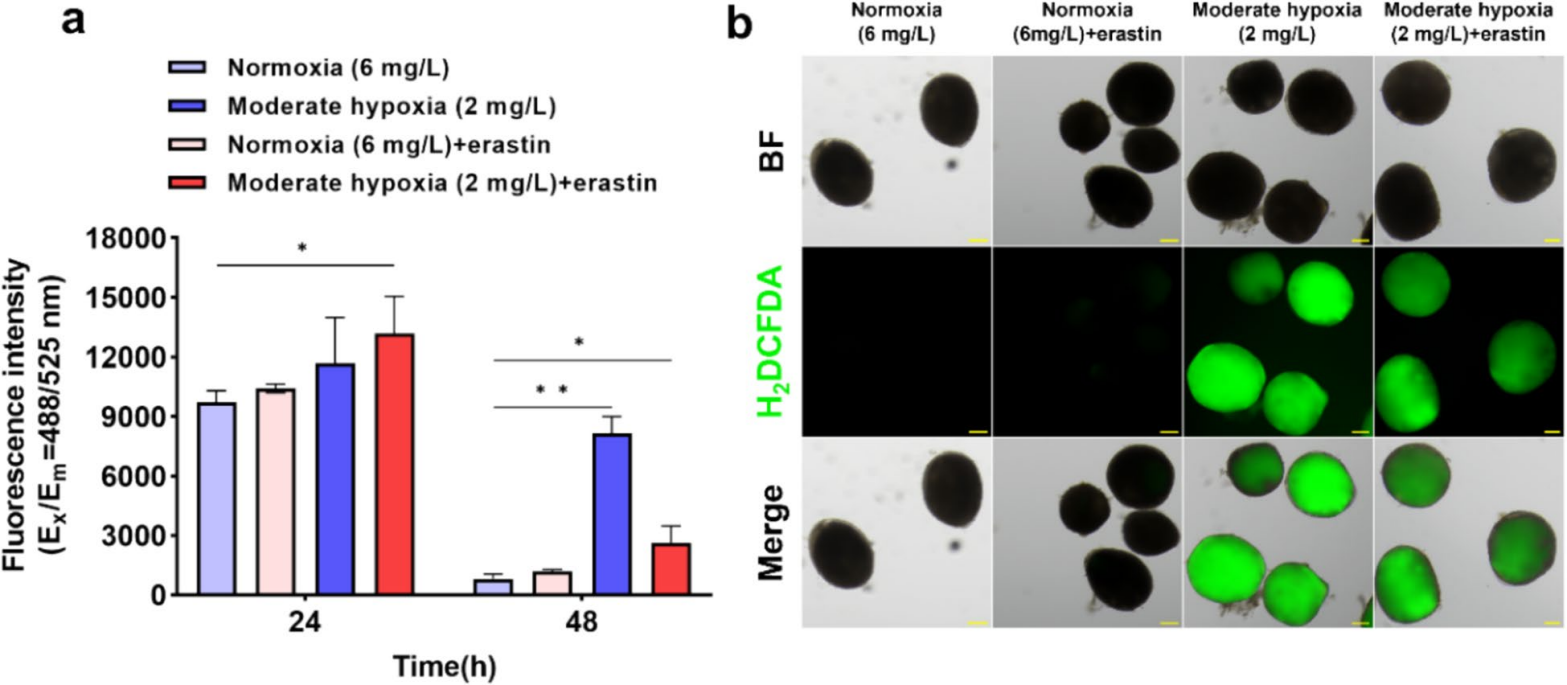
Effect of erastin treatment on reactive oxygen species (ROS) levels in *Cryptocaryon irritans* tomonts under normoxia and hypoxia (*n* = 3). **a** Fluorescence intensity at E_x_/E_m_ = 488/525 nm. **b** ROS fluorescence staining images of *C. irritans* tomonts under E_x_/E_m_ = 488/525 nm at 48 h in each group. Scale bar, 100 µm. ROS at 24 h were analyzed using the Kruskal–Wallis test followed by Dunn’s multiple-comparisons test, whereas ROS at 48 h were analyzed by one-way ANOVA with Dunnett’s multiple comparisons test. Data are shown as mean ± SD. ‘n’ indicates biological replicates. *p < *0.05* and **p < *0.01*

To evaluate hypoxia-driven oxidative stress and whether erastin amplifies it, we quantified ROS at Ex/Em = 488/525 nm across the mid and late phases. In the mid phase, ROS was relatively stable under normoxia and increased with moderate hypoxia, showing a further rise when erastin was combined with moderate hypoxia (*P* < *0.05*). By the late phase, ROS remained low under normoxia, whereas moderate hypoxia produced a marked elevation (*P* < *0.01*). Notably, adding erastin under moderate hypoxia lowered ROS relative to hypoxia alone but levels still exceeded normoxia *(P* < *0.05*) (Fig. 6a). Clear green fluorescence was observed in the moderate hypoxia (2 mg/L) and moderate hypoxia (2 mg/L) + erastin groups (Fig. 6b).

### Erastin induces mitochondrial damage, Ca^2+^ and Fe^2^⁺ release, and lipid peroxidation in *C. irritans* tomonts under hypoxia

**Fig. 7:**
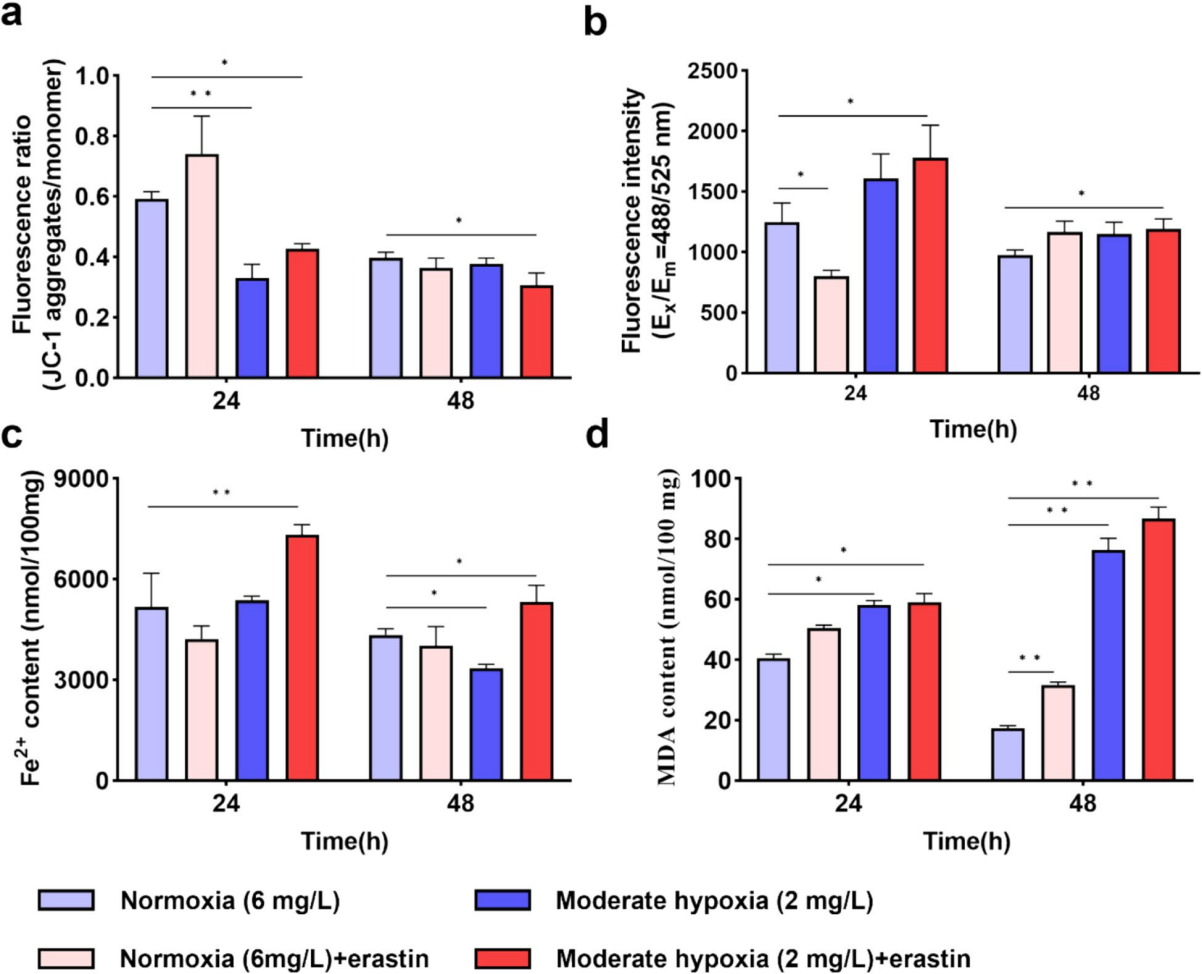
Effect of erastin treatment on **a** mitochondrial membrane potential (ΔΨm), **b** Ca^2+^ levels, **c** Fe^2+^ content and **d** MDA content in *Cryptocaryon irritans* tomonts treated with erastin under normoxia and hypoxia (*n* = 3). MDA at 24 h was analyzed using the Kruskal–Wallis test followed by Dunn’s multiple-comparisons test. All other datasets were analyzed by one-way ANOVA with Dunnett’s multiple comparisons test. Data are shown as mean ± SD. ‘n’ indicates biological replicates. *p < *0.05* and **p < *0.01*

Given the mid-to-late rise in ROS and the loss of hatching and viability under hypoxia, we quantified ferroptosis-associated changes in tomonts and evaluated amplification by erastin. The JC-1 aggregate-to-monomer ratio declined under moderate hypoxia in the mid phase and remained decreased in the late phase, with moderate hypoxia plus erastin showing the lowest values relative to normoxia (*P* < *0.05*) (Fig. 7a).

The Ca^2^⁺ level showed a phase-specific pattern. In the mid phase, erastin decreased Ca^2^⁺ under normoxia (*P* < *0.05*), whereas the moderate hypoxia (2 mg/L) + erastin group increased Ca^2^⁺ relative to normoxia group (*P* < *0.05*). Hypoxia alone did not differ from normoxia. In the late phase, only the moderate hypoxia (2 mg/L) + erastin group remained elevated versus normoxia group (*P* < *0.05*) (Fig. 7b). The Fe^2 +^ content displayed a phase-dependent divergence between hypoxia alone and hypoxia with erastin. In the mid phase, moderate hypoxia by itself did not differ from normoxia, whereas erastin increased Fe^2^⁺ under moderate hypoxia (*P* < *0.05*). By the late phase, The Fe^2 +^ contents decreased below normoxia with moderate hypoxia alone, while the moderate hypoxia (2 mg/L) + erastin group remained elevated above normoxia group (*P* < *0.05*) (Fig. 7c).

The MDA content increased under moderate hypoxia in the mid phase, with moderate hypoxia (2 mg/L) + erastin showing a comparable or greater elevation (*P* < 0.05). By the late phase, moderate hypoxia (2 mg/L) alone rose well above normoxia and moderate hypoxia combined with erastin reached the highest levels, and even normoxia (6 mg/L) + erastin group became significantly elevated relative to normoxia (6 mg/L) group (*P* < 0.01) (Fig. 7d). Taken together, the convergence of ΔΨm loss, Ca^2^⁺ perturbation, erastin-sustained elevation of Fe^2^⁺ under hypoxia, and rising MDA from the mid to late phases delineates a hypoxia-driven trajectory amplified by erastin toward ferroptotic injury.

### Erastin induces spatiotemporal changes in gene expression levels of *C. irritans* tomonts under hypoxia

**Fig. 8:**
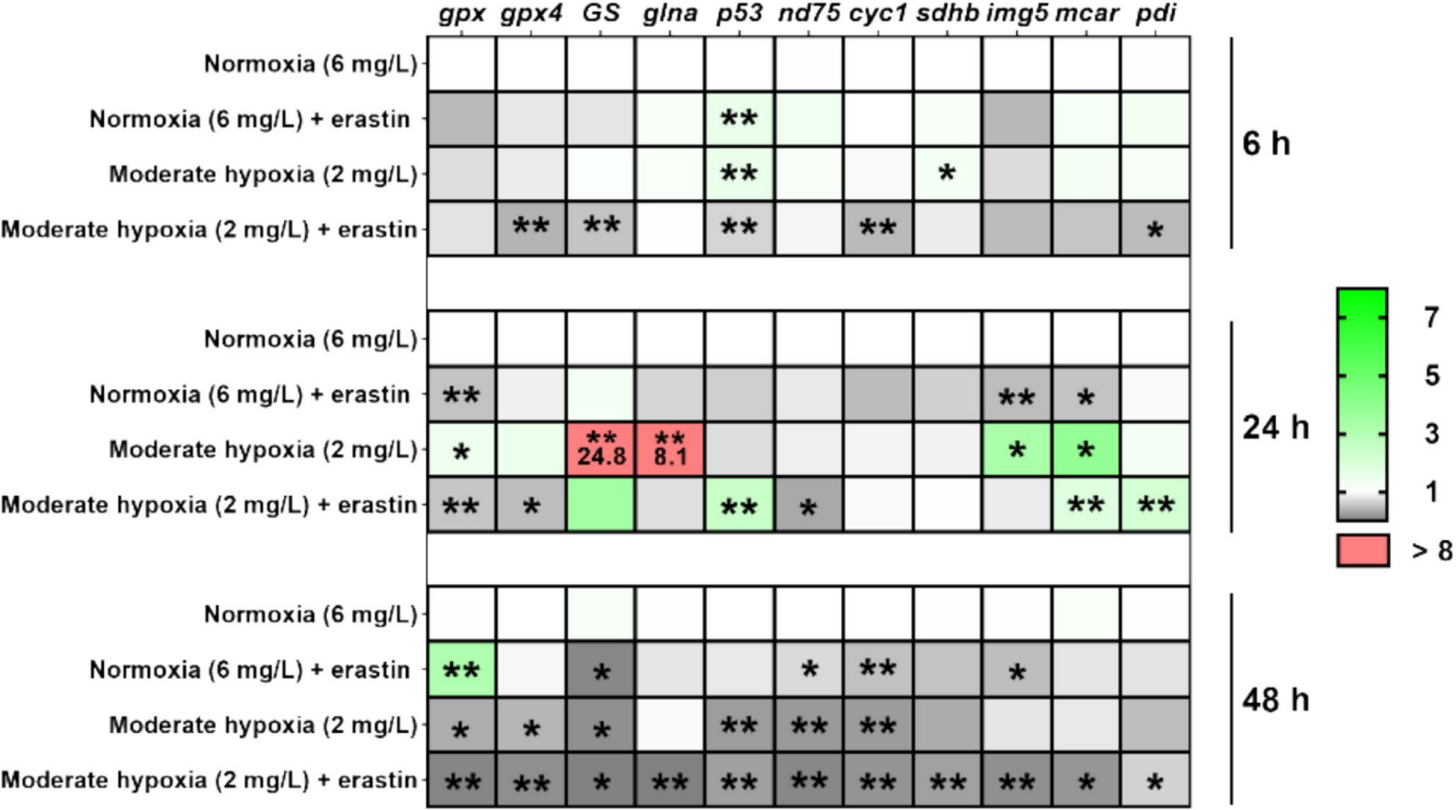
Heatmap displaying RT-qPCR expression profiles of ferroptosis genes in *Cryptocaryon irritans* tomonts (*n* = 3). Green indicates gene upregulation, grey indicates downregulation, and red highlights genes with more than 8-fold upregulation. Statistical analysis was performed using one-way ANOVA with Dunnett’s multiple comparisons test. * *p* < *0.05* and **p < *0.01*

To establish a coherent progression from hypoxia through metabolic reprogramming to ferroptotic pressure, we next examined ferroptosis-related and mitochondrial-stress transcripts under hypoxia with or without erastin. In the early phase, moderate hypoxia plus erastin showed a repressive signature that included *gpx4*, *GS*, *p53*, *cyc1*, and *pdi*, whereas moderate hypoxia alone elevated *p53* and *sdhb* (*P* < *0.05*). In the mid phase, moderate hypoxia alone prominently increased the expression levels of *GS* and *glna*, whereas erastin under moderate hypoxia selectively upregulated the expression levels of *p53*, *mcar*, and *pdi*, marking the onset of a stress-response program alongside emerging broad repression (*P* < *0.05*). In the late phase, this repression under moderate hypoxia plus erastin became widespread. In the moderate hypoxia (2 mg/L) + erastin group, the expression levels of *gpx*, *gpx4*, *GS*, *glna*, *p53*, *nd75*, *cyc1*, *sdhb*, *img5*, *mcar*, and *pdi* were significantly suppressed (*P* < *0.05*) (Fig. 8 and and Supplemental Figure S6). Taken together, the transcriptional patterns across phases, from early selective changes to mid phase induction of stress genes and culminating in late widespread repression under moderate hypoxia with erastin, delineate a transition from metabolic adjustment to ferroptotic pressure and align with the oxidative and metabolic readouts.

## Discussion

Oxygen availability shapes protozoan parasite survival and metabolism (Lloyd et al. 2021; Obirikorang et al. 2023; Prokić et al. 2025). Because hypoxia induces metabolic dysregulation, oxidative stress, and lipid peroxidation, we tested the hypothesis that ferroptosis is engaged in tomonts under hypoxia and contributes to their vulnerability (Zheng et al. 2023). Here, we interpret our data within this framework, linking hypoxia-driven metabolic and redox shifts to markers of ferroptotic pressure.

As mitochondria integrate oxygen supply with cellular metabolism, their ultrastructure provides a sensitive readout of hypoxic stress in protozoa. Mitochondria are the primary sites for ATP production and are rich in various metabolic enzymes (D’Elia et al. 2025; Harner et al. 2016). In protozoa, mitochondrial cristae are tubular (Han et al. 2023; Sukhorukov and Bereiter-Hahn 2009) and can be lost by hypoxia, which also results in a darker mitochondrial matrix, denser packing of cristae, as well as mitochondrial swelling and near-spherical morphology (Mironova et al. 2019; Morozov and Rakic 2023; Qiu et al. 2024). In this study, hypoxia caused cellular swelling and vacuolization of mitochondria in *C. irritans* tomonts. These findings set the stage for subsequent sections that interrogate hypoxia-driven metabolic and redox perturbations and evaluate evidence for ferroptotic pressure.

Anchored by hypoxia-induced mitochondrial ultrastructural lesions, we next interrogated the organism’s energetic and redox responses. During hypoxia, energetic and redox readouts indicate a biphasic trajectory. *C. irritans* does not uniformly up-regulate glycolysis; rather, it exhibits phasic regulation over time. An early glycolytic compensation, initiated by HK-mediated phosphorylation of glucose as glucose-6-phosphate and by cytosolic NAD⁺ regeneration via lactate dehydrogenase, limits immediate dependence on mitochondrial oxidative phosphorylation (OXPHOS). Later, an increase in PK activity reinforces terminal substrate-level phosphorylation once a new redox steady state is established (DeMichele et al. 2024; Mai et al. 2015; Roberts and Miyamoto 2015; Schormann et al. 2019). This phased response smooths glycolytic flux, mitigates potential bottlenecks and futile cycling, and supports ATP economy under sustained hypoxia. However, as substrate availability and redox buffering wane, ATP levels and the NAD⁺/NADH balance decline, pyruvate is increasingly routed to lactate, and oxidative stress intensifies, linking ultrastructural mitochondrial damage and electron-transport impairment to metabolic failure (Berthiaume et al. 2019; Fan et al. 2024; Tang et al. 2024). Functionally, this transient compensatory window likely helps tomonts meet basal energy demands under benthic hypoxia long enough to progress through encystment and hatching, whereas prolonged hypoxia erodes this buffer and compromises viability (Wang et al. 2025).

Finally, we use metabolite-supplementation assays to probe pathway constraints and metabolic plasticity under low oxygen. The metabolite supplementation assay further showed that, under hypoxia, *C. irritans* maintains substrate-level phosphorylation by up-regulating the entry enzyme HK and the terminal enzyme PK at early time points, but with prolonged hypoxia it shifts to a less efficient fermentation fallback, revealing a downstream bottleneck (DeMichele et al. 2024; Schormann et al. 2019). Mechanistically, isomaltose likely supplements glucose after hydrolysis by α-glucosidase, thereby aligning with early HK reinforcement (Bravo-Torres et al. 2004). Mannitol and L-histidine may act as compatible solute/buffering factors to lower osmotic and acid–stress costs, helping to sustain function when the pathway endpoint is constrained (Abe 2000; Ruijter et al. 2003). The effect of isovalerate is consistent with ACSS up-regulation, suggesting that an acetate/acetyl-CoA bypass helps buffer carbon flux (Schug et al. 2015). In contrast, glutamine/carnitine/citrulline primarily engage aerobic pathways or nitrogen metabolism, which are not the current bottlenecks, and therefore show no rescue effect (Imbard et al. 2023; Longo et al. 2016; Yang et al. 2014). Overall, these supplementation results reveal a metabolically plastic parasite that can be “leveraged” by exogenous metabolites—carbon-flux supply and stress-cost reduction cooperate to maintain energy and homeostasis in the short term, while simultaneously exposing its dependence on the fermentation endpoint and on acetyl-CoA supply.

Having delineated pathway constraints and metabolic plasticity under low oxygen, we next consider how this milieu conditions vulnerability to ferroptotic stress, with erastin serving solely as a pharmacologic probe that reveals and amplifies such liability (Du and Guo 2022; Stockwell and Jiang 2020). Hypoxia creates a more fragile homeostatic state for tomonts; even after returning to normoxia, tomonts hatching rates fail to recover to normal levels. As a ferroptosis inducer, erastin further amplifies this vulnerability under hypoxia, leading to an additional decline in hatching and manifesting signs of impaired membrane homeostasis, including enlarged intercellular spaces and plasma-membrane damage (Wang et al. 2025).

In protozoan parasitology, excessive ROS can precipitate ferroptotic cell death (Li et al. 2022). Ferroptosis is often initiated by disruption of intracellular redox homeostasis (He et al. 2021). In this study, we observed a marked increase in intracellular ROS at 24 h under combined erastin and hypoxia treatment, indicating that erastin under hypoxia markedly promotes this process. Erastin may suppress cystine uptake via inhibition of system xc⁻, thereby limiting glutathione biosynthesis, while hypoxia itself diminishes the cell’s redox-buffering capacity (Sato et al. 2018). The conjunction of these mechanisms may overwhelm primary antioxidant defenses and precipitate an initial surge of ROS (Smith et al. 2017). At later time points, ROS signals declined in some groups. Rather than a simple waning of signal, this dynamic likely reflects a transition in cell fate during the death program, suggesting passage beyond an irreversible injury threshold and progression toward a terminal stage characterized by membrane system failure (Sato et al. 2018; Schieber and Chandel 2014).

One of the principal targets of the initial ROS surge is the mitochondrion (He et al. 2021; Zheng et al. 2023). In our study, combined erastin and hypoxic stress was associated with a collapse of the ΔΨm, a hallmark of compromised core mitochondrial function (He et al. 2021). We infer that this reflects not only passive ROS-mediated injury but also a more programmatic process (Schieber and Chandel 2014). For example, erastin has been proposed to modulate channels such as VDAC, thereby promoting mitochondrial membrane permeabilization (Gao et al. 2019). Loss of ΔΨm directly disrupts the mitochondrial contribution to cellular ionic homeostasis, facilitating efflux of matrix Ca^2^⁺ into the cytosol and, given the organelle’s role in cellular iron handling, perturbing Fe^2^⁺ dynamics linked to iron–sulfur cluster metabolism or iron storage. Released Fe^2^⁺ can sustain further ROS generation through Fenton-type chemistry, whereas Ca^2^⁺ accumulation may activate phospholipases and disturb membrane asymmetry, together creating conditions that favor lipid peroxidation (De Stefani et al. 2015; Maher et al. 2018; Wang et al. 2020; Zheng et al. 2023).

Driven by ROS, labile Fe^2^⁺, and allied drivers, polyunsaturated fatty acids undergo lethal lipid peroxidation, which constitutes the terminal execution phase of ferroptosis (Li et al. 2022; Tang et al. 2021). In tomonts subjected to combined erastin and hypoxia, MDA levels rose markedly and remained persistently elevated, indicating strong activation of lipid peroxidation and irreversible injury to the tomonts. This process directly drives tomonts toward ferroptotic death.

*C. irritans* poses a major threat to marine fish, and recent efforts increasingly focus on enhancing host resistance through vaccine development, selective breeding for disease resistance, and the use of protective materials (Wu et al. 2025; Zhong et al. 2021). However, parasite-centered approaches, particularly those targeting specific cell death pathways, remain underexplored. Previous studies postulate that drugs and metals can induce apoptosis-like death and cuproptosis in tomonts (Siddique et al. 2025; Zhao et al. 2023). In this study, we demonstrated for the first time that hypoxia induces ferroptosis in *C. irritans* tomonts—a non-apoptotic, iron-dependent cell death characterized by oxidative stress, lipid peroxidation, and mitochondrial dysfunction. Although *C. irritans* is known to respond to environmental variables such as DO (Wang et al. 2025; Watanabe et al. 2018), temperature (Yin et al. 2023) and salinity (Kong et al. 2022), the mechanisms by which it adapts—or succumbs—to such stressors remain poorly understood. Our findings show that hypoxia is a potent inducer of ferroptosis in tomonts, while in our preliminary experiments, extreme temperature and salinity stress failed to trigger ferroptosis even in the presence of erastin. This suggests that ferroptosis in *C. irritans* is not a general stress response but a specific vulnerability linked to redox imbalance and oxygen deprivation. Future studies should explore whether ferroptosis-inducing compounds can be safely applied in aquaculture systems and whether similar mechanisms exist in other protozoan parasites. Elucidating the ecological roles and evolutionary conservation of ferroptosis across diverse parasitic lineages may provide valuable insights into parasite control strategies and stress adaptation in marine environments.

## Conclusion

Hypoxia drives pronounced metabolic reprogramming and mitochondrial dysfunction in *C. irritans* tomonts. Erastin further exacerbates these effects, culminating in ferroptosis marked by elevated ROS, lipid peroxidation, and Ca ⁺/Fe ⁺ imbalance. Together, these findings identify a previously underappreciated regulated cell-death pathway in marine parasitic ciliates and point to actionable targets for developing new control strategies against *C. irritans*.

## Materials and methods

### Fish, *C. irritans* isolation and propagation

Pompano weighing 500 ± 100 g were reared at the Daya Bay Aquaculture Experimental Center in Huizhou City, Guangdong Province, China (Water: 26-28 °C, Salinity: 27–32 ‰, DO: 5–6 mg/L) for use as the experimental fish. A local strain of *C. irritans* was isolated from pompano obtained from an aquafarm in Huizhou City, Guangdong Province, China (Dan et al. 2006). The fish were relocated to clean-bottomed tanks following the observation of white spots on the pompano. The tomonts were collected from the tank bottom after the trophonts detached and were subsequently thoroughly washed with sterile seawater before being cultured at 26-28 °C. Theronts began to be released from the tomonts after 72 h and were collected within 2 h. The theronts were then reintroduced into the Pompano tanks for reinfection (Dan et al. 2006).

### Experimental design and grouping

**Fig. 9:**
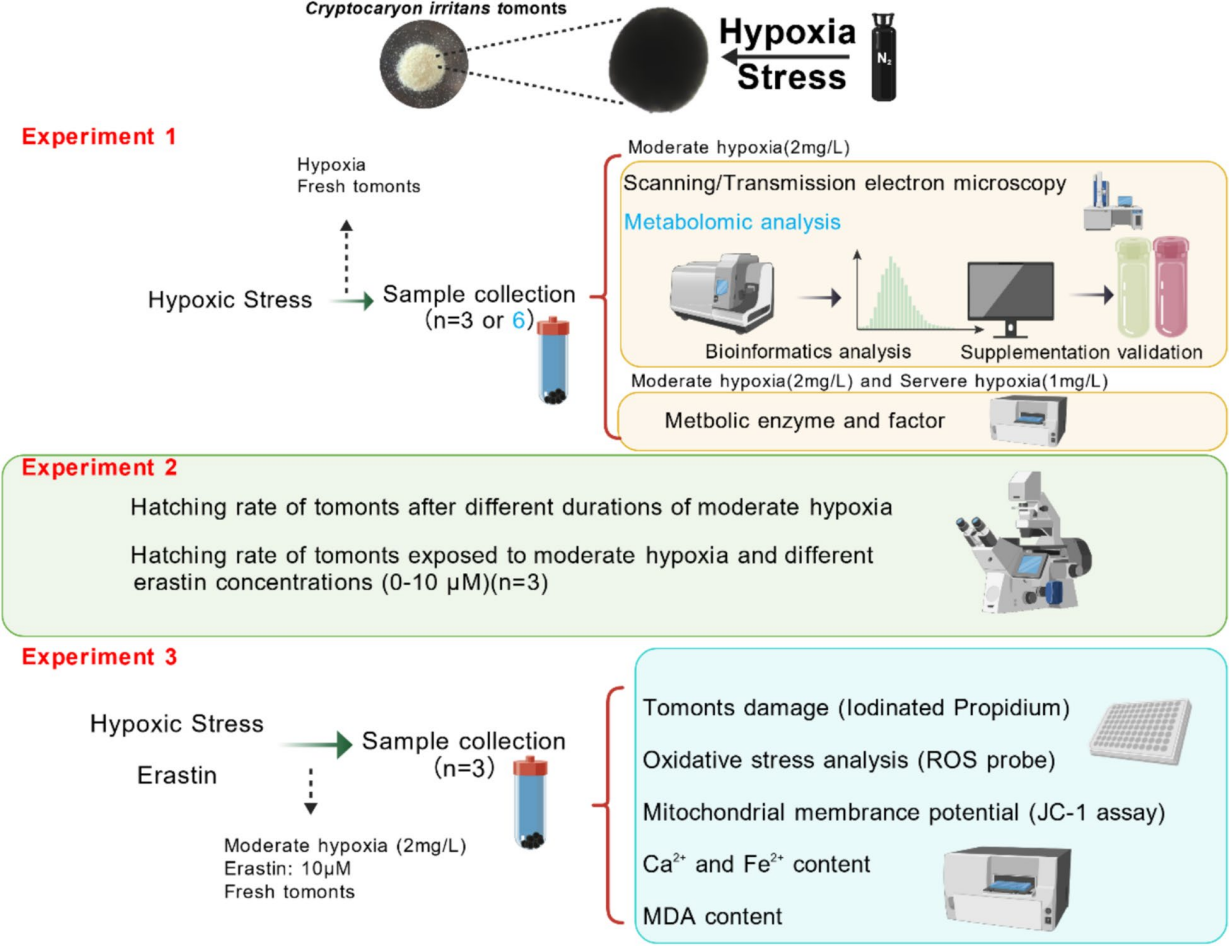
Experimental workflow

Tomonts of *C. irritans* were collected 10–12 h after trophonts detached from the host and were washed three times with sterilized seawater. The study was conducted in three sequential experimental phases to investigate the impact of hypoxia and to assess the potential involvement of ferroptosis in hypoxia-induced damage (Fig. 9).

#### Experiment 1

This phase focused on evaluating the physiological and metabolic responses of tomonts under hypoxic conditions. The hypoxia induction method was adapted from Wang et al. (2025). DO concentrations in seawater were continuously monitored using an oxygen meter (SMART SENSOR, AR8406, China). Nitrogen gas (N₂) was bubbled into seawater until the DO levels reached 2 and 1 mg/L, which were designated as the moderate hypoxia (2 mg/L) group and severe hypoxia (1 mg/L) group, respectively (Roman et al. 2019). Once the desired DO levels were achieved, nitrogen bubbling was immediately halted, and tomonts were transferred into the hypoxic seawater. Seawater with a DO concentration of 6 mg/L was used as the normoxia (6 mg/L) group. Metabolome, enzyme activities, and ultrastructural alterations of *C. irritans* tomonts were examined under hypoxia.

#### Experiment 2

First, we measured the hatching rate of tomonts after different durations of moderate hypoxia (2 mg/L) followed by reoxygenation (normoxia: 6 mg/L). Second, tomonts were exposed to moderate hypoxia (2 mg/L) with or without erastin. A pilot erastin dose-finding assay under moderate hypoxia was conducted to determine a working concentration.

#### Experiment 3

10 µM erastin was selected and applied for all endpoints. Four experimental groups were established: the normoxia (6 mg/L) group, the normoxia (6 mg/L) + erastin group, the moderate hypoxia (2 mg/L) group and the moderate hypoxia (2 mg/L) + erastin group. Treatment solutions were freshly prepared in seawater equilibrated to the corresponding DO levels and distributed into sterile centrifuge tubes. After triple washing, tomonts were transferred into the treatment tubes, and excess seawater was gently removed to ensure consistent exposure. All transfers were completed swiftly to minimize oxygen fluctuations and mechanical stress. ROS production, MDA content and Ca^2^⁺ and Fe^2^⁺ dynamics in *C. irritans* tomonts were evaluated under hypoxia and erastin exposure.

### Scanning electron microscope (SEM) and Transmission electron microscopy (TEM) observation of the morphology

Tomonts were collected 24 h after hypoxia stress. They were placed in 2.5% glutaraldehyde solution, immediately vacuumed, and stored at 4 ℃ until sample preparation. Briefly, SEM samples were washed three times with 0.1 M Phosphate buffered saline (PBS) and dehydrated through a graded ethanol series (30, 50, 70, 80, 90, and 100%), each for 15 min. Samples were then dried using a Tousimis SAMDRI-PVT-3D critical point dryer (Tousimis, Maryland, USA) with liquid CO₂ and mounted onto stubs using conductive carbon adhesive tabs. The tomonts were examined using an S–3400N scanning electron microscope (Hitachi, Tokyo, Japan) at an accelerating voltage of 10 kV. TEM samples were washed with 0.1 M PBS and fixed with 1% osmium tetroxide solution at room temperature for 1.5 h. The fixed samples were dehydrated through graded ethanol, infiltrated, and embedded in acetone and resin. Continuous ultrathin sections were cut using an ultrathin sectioning system (Leica EM UC7, Wetzlar, Germany). Sections were stained with uranyl acetate and lead citrate to resolve mitochondrial ultrastructure, after mounting on slot grids and examined in a JEM-1400Flash TEM (JEOL Ltd., Tokyo, Japan) at an accelerating voltage of 120 kV (Li et al. 2024; Ma et al. 2017).

### Metabolite extraction, LC–MS/MS analysis, and validation of metabolite supplementation function

Metabolomic analysis was also performed 24 h after hypoxia stress with six biological replicates. Approximately 50 mg of tomonts were collected into cryovial, immediately frozen in liquid nitrogen and stored at −80 °C. The tomonts were mixed with beads and 500 μL of extraction solution, consisting of methanol, acetonitrile and H_2_O, prepared in the ratio of 2:2:1 (v/v/v). The mixture was vortexed for 30 s, ground at 35 Hz for 4 min, and then subjected to ultrasonication in an ice-water bath for 5 min in triplicates. After incubation at −40 °C for 1 h, the sample was centrifuged at 12,000 rpm for 15 min at 4 °C. The supernatant was transferred to a vial for instrumental analysis, and equal amounts from all samples were pooled to prepare QC samples.

For polar metabolites, LC–MS/MS analyses were performed using a UHPLC system (Vanquish, Thermo Fisher Scientific) with a Waters ACQUITY UPLC BEH Amide (2.1 mm × 50 mm, 1.7 μm) coupled to an Orbitrap Exploris 120 mass spectrometer (Orbitrap MS, Thermo). The mobile phase consisted of 25 mmol/L of each ammonium acetate and ammonia hydroxide in water (pH = 9.75) (A) and acetonitrile (B). The auto-sampler temperature was 4 ℃, and the injection volume was 2 μL. The Orbitrap Exploris 120 mass spectrometer was used due to its ability to acquire MS/MS spectra in information-dependent acquisition mode in the control of the acquisition software (Xcalibur, Thermo). In this mode, the acquisition software continuously evaluates the full scan MS spectrum. The ESI source conditions included a sheath gas flow rate of 50 Arb, aux gas flow rate of 15 Arb, capillary temperature of 320 ℃, full MS resolution at 60,000, MS/MS resolution at 15,000, collision energy: SNCE 20/30/40, spray voltage at 3.8 kV (positive) or −3.4 kV (negative). The raw data were converted to the mzXML format using ProteoWizard and processed with an in-house program developed using R and based on XCMS, for peak detection, extraction, alignment, and integration. The R package and the BiotreeDB (V3.0) were applied in metabolite identification as described previously (Zhou et al. 2022).

The eight differential metabolites, including L-histidine, N-acetyl-β-alanine, mannitol, isomaltose, glutamine, citrulline, isovaleric acid, and L(-)-carnitine that decreased after hypoxic exposure to *C. irritans* tomonts were selected for supplementation experiments with three biological replicates. About 40 to 60 tomonts were placed in hypoxic seawater containing 100 µmol/L solution of each selected metabolite. The tomonts were then incubated at 26 °C for 7 days, and the final hatching rate was calculated using the following formula: The hatching rate = 100% × number of tomonts hatched/total number of tomonts as described previously (Wang et al. 2025; Zhong et al. 2022).

### Determination of metabolite concentrations

Antioxidant enzyme levels and metabolic enzyme activities with three biological replicates were measured at 6, 24, and 48 h after hypoxia in fresh tomonts by determining the contents of MDA (Solarbio, BC0025, China), and the activities of CAT (Solarbio, BC0205, China), HK (Solarbio, BC0745, China) and PK (Solarbio, BC0540, China) with their respective enzyme assay kits. The energy parameters were measured using the coenzyme I NAD^+^/NADH content and ATP content assay kit (Biosharp, BL1482A, China), while lactate content was measured after 48 h of hypoxia in tomonts using the lactic acid content assay kit (Biosharp, BL868B, China).

### Hatching rate of tomonts following different durations of hypoxia exposure

Fresh tomonts (20–40) were transferred into 5 mL centrifuge tubes containing normoxic or hypoxic seawater with three biological replicates. Tomonts in the hypoxia group were exposed to DO 2 mg/L for 6 h, 1, 2, 3, and 7 d. The tomonts were subsequently transferred into 48-well plates containing normoxic seawater (DO 6 mg/L) after each exposure period. The hatching rate (%) was calculated using the following formula: 100 × (number of tomonts hatched/total number of tomonts) (Wang et al. 2025).

### Effect of erastin treatment on tomonts’ hatching rate

Erastin was added to both the normoxia and hypoxia groups at final concentrations of 0, 2, 4, 8, and 10 μM with three biological replicates. The tomonts were incubated at 26 °C for 8 days, after which the hatching rate was calculated using the formulae below. Morphological observations of tomonts in the four groups were subsequently made using an inverted microscope (Nikon Eclipse Ti2-E, Japan).

### PI staining

Tomonts were transferred to 5 mL centrifuge tubes and subjected to hypoxia and 10 μM erastin treatment with three biological replicates. The tomonts were then washed three times with PBS (pH = 7.4, 0.01 mol/L) and resuspended after 48 h of stress. Tomonts were stained with PI following the manufacturer’s instructions (Guangzhou Huayun biotech Co.,LTD, HYPC5268). The tomonts were then incubated in 10 μM PI solution at 26 °C for 20 min and subsequently washed thrice with PBS. One hundred tomonts per well were transferred into a 48-well plate, followed by measurement of the fluorescence intensity using a microplate reader (BioTek H1MF, BioTek Instruments, Inc., USA) with E_x_/E_m_ set at 535/615 nm. The staining results were observed under an inverted fluorescence microscope (Nikon Eclipse Ti2-E, Nikon Instruments Inc., Japan).

### ROS quantification

Given that *C. irritans* tomonts exceed 200 μm and are relatively opaque, rendering conventional flow cytometry unsuitable, we assessed fluorescence using optical density based measurements instead (Zhao et al. 2023). The ROS levels in tomonts from each group were assessed 24 and 48 h post-treatment with three biological replicates. The tomonts were washed three times with PBS and subsequently incubated with 10 μM H_2_DCFDA at 26 ℃ for 30 min (Biosharp, BL714A, China). The tomonts were then washed thrice with PBS. One hundred tomonts per well were transferred into a 48-well plate, followed by measurement of the fluorescence intensity using a microplate reader (BioTek H1MF, BioTek Instruments, Inc., USA) with E_x_/E_m_ set at 488/525 nm. The fluorescence of the tomonts at 48 h was captured using an inverted fluorescence microscope (Nikon Eclipse Ti2-E, Nikon Instruments Inc., Japan).

### Analysis of mitochondrial membrane potential, Ca^2^⁺, Fe^2^⁺, and MDA

The ΔΨm in *C. irritans* tomonts was determined using a mitochondrial membrane potential assay kit with JC-1 (Biosharp, BL711A, China) with three biological replicates. The tomonts were treated with JC-1 working solution at 26 °C for 20 min and then washed twice with JC-1 staining buffer (1 ×). A total of 100 tomonts per well were transferred to a 48-well plate, followed by the detection of JC-1 monomers and JC-1 aggregates at E_x_/E_m_ of 490/530 nm and 525/590 nm, respectively, using a microplate reader (BioTek H1MF, BioTek Instruments, Inc., USA).

The Ca^2+^ concentration of the tomonts was determined using a Fluo-3 AM calcium concentration detection kit (Solarbio, M8650, China) with three biological replicates. Tomonts were incubated with 3 μM Fluo-3 AM solution prepared in HBSS buffer at 26 °C for 20 min, after which 5 volumes of HBSS containing 1% fetal bovine serum was added and incubation continued for 40 min. Tomonts were washed thrice with HEPES-buffered saline solution. Their fluorescence intensity was then measured using a microplate reader (BioTek H1MF, BioTek Instruments, Inc., USA) with E_x_/E_m_ set at 488/525 nm. The Fe^2+^ and MDA content in the tomonts was determined using a Ferrous Ion Content Assay Kit and MDA Content Assay Kit at 24 and 48 h post-treatment according to the manufacturer’s instructions (Biosharp, BL1147B and Solarbio, BC0025, China) with three biological replicates.

### Quantitative real-time PCR (qRT-PCR) analysis

Total RNA from the post-treated tomonts was extracted using the animal total RNA isolation kit (Foregene, Guangzhou Weidexin Biotechnology Co., Ltd.) following the manufacturer’s protocol with three biological replicates. The quantity, purity, and integrity of the extracted RNA were assessed. The RNA was then reverse transcribed into cDNA using the Evo M-MLV RT Mix Kit with gDNA Clean for qRT-PCR Ver.2 (Accurate Biotechnology, Hunan, Co., Ltd.) and stored at − 20 °C. The primers used for qRT-PCR (Supplemental Table S1) were designed using the Primer3Plus (https://www.primer3plus.com), while *EF-1β* was used as the internal reference gene. The same qRT-PCR workflow was applied to two predefined gene sets: metabolism-related genes and ferroptosis-associated genes. Relative transcript levels were calculated using the 2^ − ΔΔCt method (Mo et al. 2016).

### Statistical analysis and image processing

In Experiments 1 and 2, differential metabolite screening, metabolite-supplementation analyses, and hatching rates under moderate hypoxia across different erastin concentrations were evaluated using two-tailed Student’s t-tests. Normality and homogeneity of variance tests were first conducted for the other data sets. One-way analysis of variance (ANOVA) followed by Dunnett’s multiple comparisons test was performed using Statistical Package for the Social Sciences (SPSS 29) when both normality and homogeneity were met. The Kruskal–Wallis test, followed by multiple comparisons using Dunn’s test, was performed when normality and homogeneity were not met. Data were presented as means ± standard deviation (SD). *P < 0.05, **P < 0.01. Data were visualized using GraphPad Prism 8. The graphical abstract and experimental workflow were generated using the GDP—Generic Diagramming Platform (Jiang et al. 2025). The fluorescent images were processed using ImageJ (ImageJ 1.54i).

## Supplementary Information

Supplementary Material 1.

## Data Availability

Data will be made available on request.

## Abbreviations

HIF: Hypoxia-Inducible Factor
DO: Dissolved Oxygen
ROS: Reactive Oxygen Species
TCA: Tricarboxylic Acid Cycle
ETC: Electron Transport Chain
MDA: Malondialdehyde
CAT: Catalase
HK: Hexokinase
PK: Pyruvate Kinase
SEM: Scanning electron microscope
TEM: Transmission electron microscoe
ATP: Adenosine Triphosphate
PI: Propidium Iodide
NAD⁺: Nicotinamide Adenine Dinucleotide
NADH: Nicotinamide Adenine Dinucleotide (Reduced form)
PBS: Phosphate-Buffered Saline
SD: Standard deviation
ANOVA: Analysis of Variance
