# Supplementary material for: Hypoxia-induced oxidative stress and mitochondrial damage initiate ferroptosis in Cryptocaryon irritans, a protozoan parasite of marine fish
Source: Stress Biol. 2026 Jan 12;6(1):6. doi: 10.1007/s44154-025-00275-0 (PMC12791103; doi:10.1007/s44154-025-00275-0)
Supplement: Supplementary file 1 — Supplementary Material 1. [file 44154_2025_275_MOESM1_ESM.docx]

**Supporting Information**

Article title: **Hypoxia-induced oxidative stress and mitochondrial damage initiate ferroptosis in *Cryptocaryon irritans*, a protozoan parasite of marine fish**

Author: Baotun Wang, Zhi Luo, Jingyu Zhuang, Zhicheng Li, Xueli Lai, Huicheng Wu, Qing Han, Jizhen Cao, Hebing Wang, Chuanfu Dong, Anxing Li

**Table Legends**

**Supplemental Table S1** Primers in this study

| prime | Sequences (5’-3’) |
| --- | --- |
| *EF-1β*-F | GGAGATGATGATGATAATGATGA |
| *EF-1β*-R | CCAAACTAAACCTTCCAACT |
| *ADP_GK*-F | TCAGTGATGCTAGGAGGACC |
| *ADP_GK*-R | TGATGGTGCTGTTATTGATCCCA |
| *pgk*-F | TGGAGTGGATTGGACTGTGG |
| *pgk*-R | ACTAGCTCCTCCTCCGGTAC |
| *acss*-F | CCAACCAGTTTCAGTTTGCCA |
| *acss*-R | CAAGGACCCAGATGCAGGAA |
| *GAPDH*-F | GTTATCATCTCAGCCCCACCT |
| *GAPDH*-R | AGTCCCTCAACAAGTCCGAA |
| *eno*-F | AGCAGCTACATCCATACCCA |
| *eno*-R | TCGGTGCTGAGGTTTACCAC |
| *camk*-F | TTGGTCATGTGGCGTGGTTA |
| *camk*-R | TGGTTGAGACATTCTTAGGCGT |
| *mtor*-F | GCTTTCGGTTCCTGGGCTAT |
| *mtor*-R | ACTCGTTCATCTTGGCGGAG |
| *glna*-F | ATGCTGAAGTTGCTCCTGGG |
| *glna*-R | CCTTCACTTCTGGTTTTGGTGG |
| *GPI1*-F | TTCAGGTTTCAACGCCAGAG |
| *GPI1*-R | TGGCCATTAGTACCAGGTTCA |
| *gpx*-F | GCTCTTCCGAATTCCTTTTCAGA |
| *gpx*-R | GCCTCTGATAATCCCTTGTCGA |
| *gpx4*-F | AAAAGACTACGAGGATTTGGGTG |
| *gpx4*-R | TGGCTCTTAACCAATGAAAAATTGA |
| *GS*-F | AGCCAGTGCCAAGATTAGGA |
| GS-R | AGCCACTCCTCCTTCATTAG |
| p53-F | ACTCCTTCCAAGCCATCACG |
| p53-R | CCACCCCAACATCCTGTCTC |
| nd75-F | AGGAGCTTCGAACCTTGGATT |
| nd75-R | GCCATCATAGGAGAATTTGCCG |
| cyc1-F | TGGGCTGGATTGATTGAGAACA |
| cyc1-R | TGAGGTCGGAACAGAACATGG |
| sdhb-F | CATGTCCTAGTTATTGGTGGCA |
| sdhb-R | TCCTCTGCAACTTTGTTCTCTCT |
| img5-F | TCAGCCCGTTTTTATCTTTCAC |
| img5-R | GTTCTTATGGAGCAATGGAACCTTT |
| mcar-F | CCCTTAGTCAGTAGCCTTTGTGA |
| mcar-R | AGGGAGGAGGGTCTTAAAGGA |
| pdi-F | TGGCCATACAATTCTGACAGT |
| pdi-R | TCGGAGAAAACAAAGCTTGCC |

**Figure Legends**


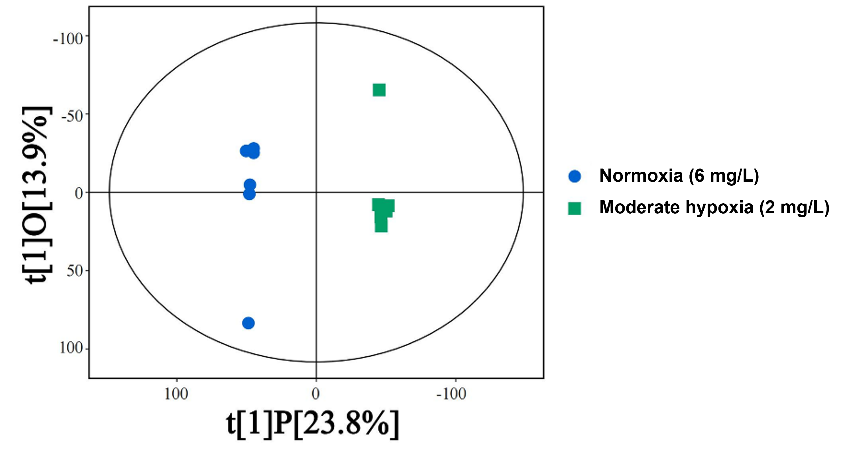


**Supplemental Figrue S1** OPLS-DA analysis of *C. irritans* tomonts under hypoxia


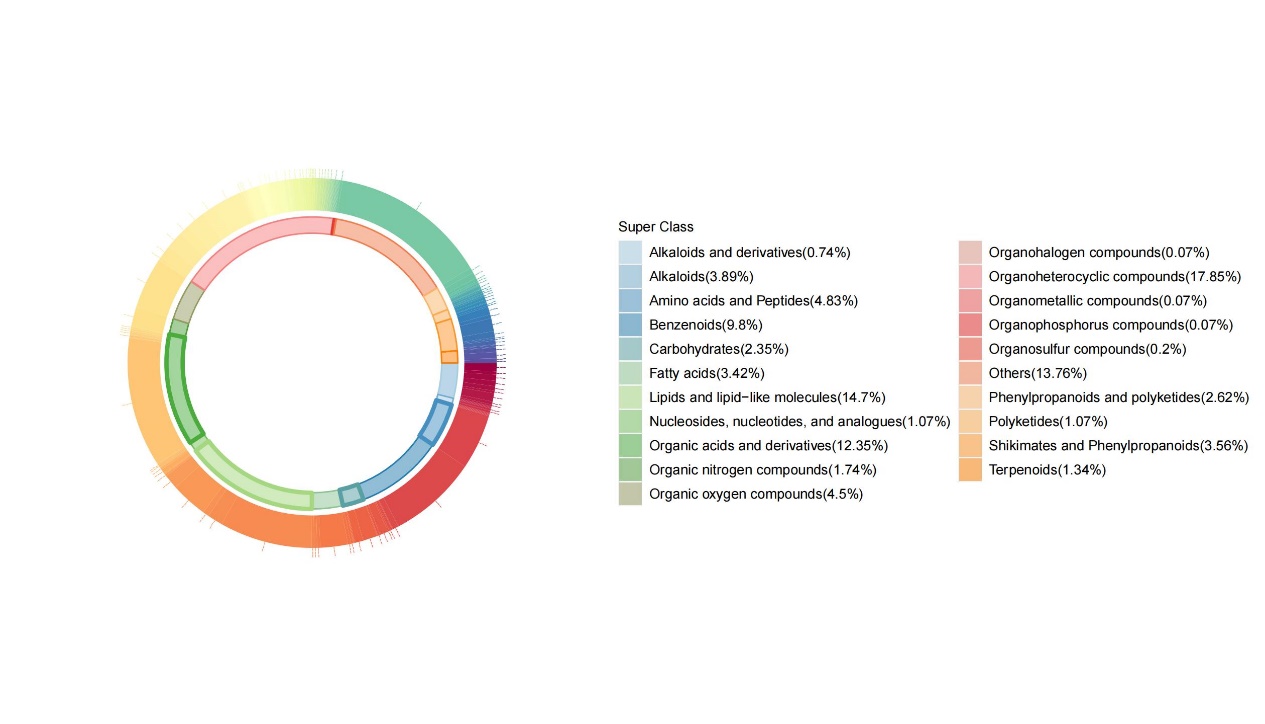
**Supplemental Figrue S2** Ring diagram shows differential metabolites in *Cryptocaryon irritans* tomonts under hypoxia

**
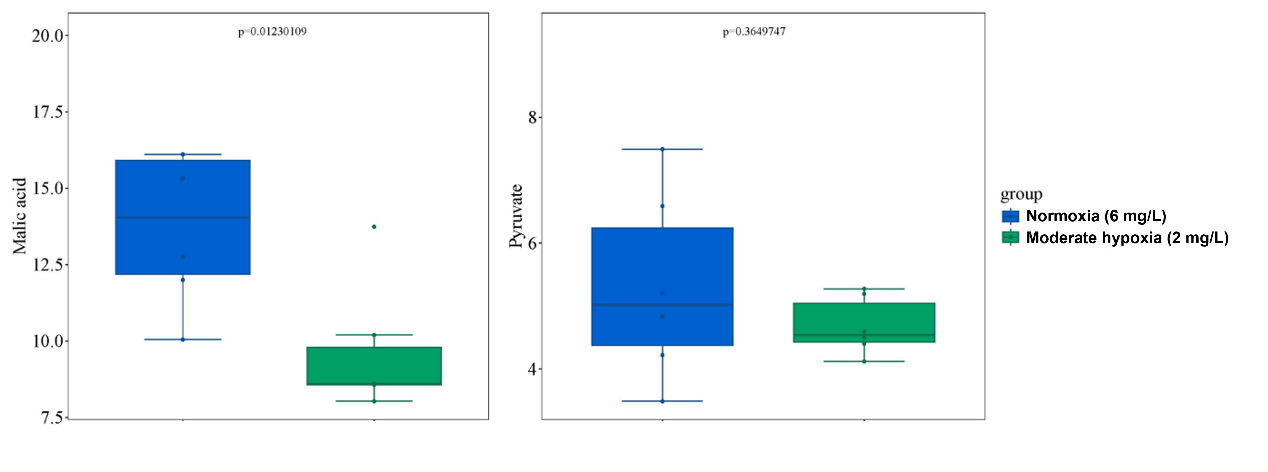
**

**Supplemental Figrue S3** Malic acid and pyruvate content in *Cryptocaryon irritans* tomonts under hypoxia. Statistical analysis in was performed using a two-tailed Student’s t-test (n=6), Data are shown as mean ± SD. ‘n’ indicates biological replicates.


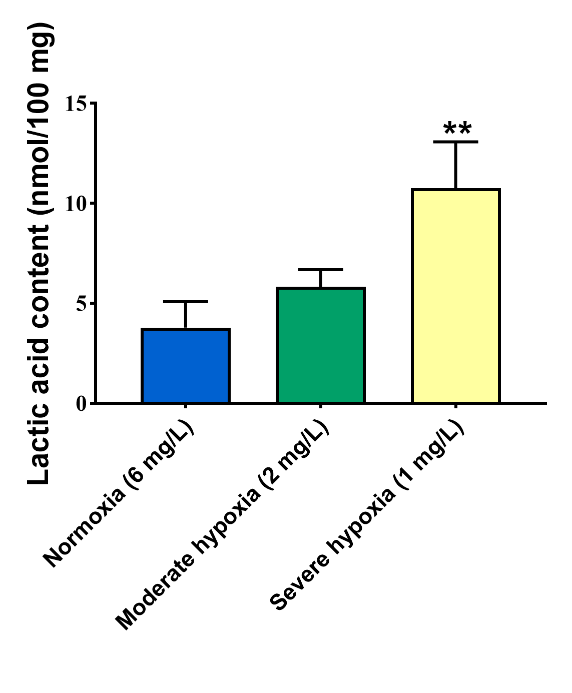


**Supplemental Figrue S4** Lactic acid content in *Cryptocaryon irritans* tomonts under hypoxia. Statistical analysis was performed using one-way ANOVA with Dunnett's multiple comparisons test (n=3). Data are shown as mean ± SD. ‘n’ indicates biological replicates. ***p<0.01*.


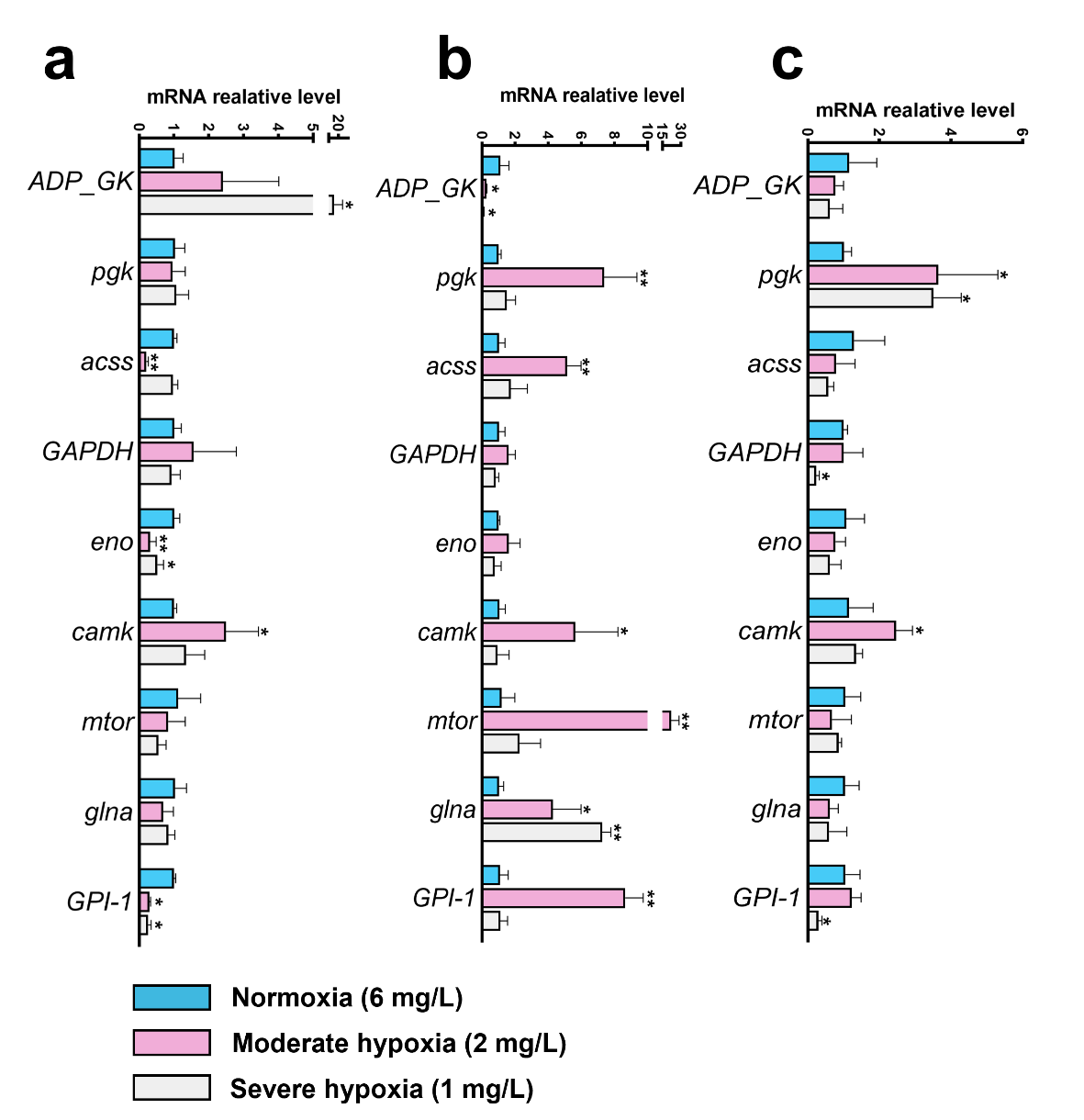


**Supplemental Figrue S5.** mRNA expression of ferroptosis-related genes in *Cryptocaryon irritans* tomonts under hypoxia. (a) 6 h, (b) 24 h, and (c) 48 h post-treatment (n=3). Statistical analysis was performed using one-way ANOVA with Dunnett's multiple comparisons test. Data are shown as mean ± SD. ‘n’ indicates biological replicates. **p<0.05* and ***p<0.01*.


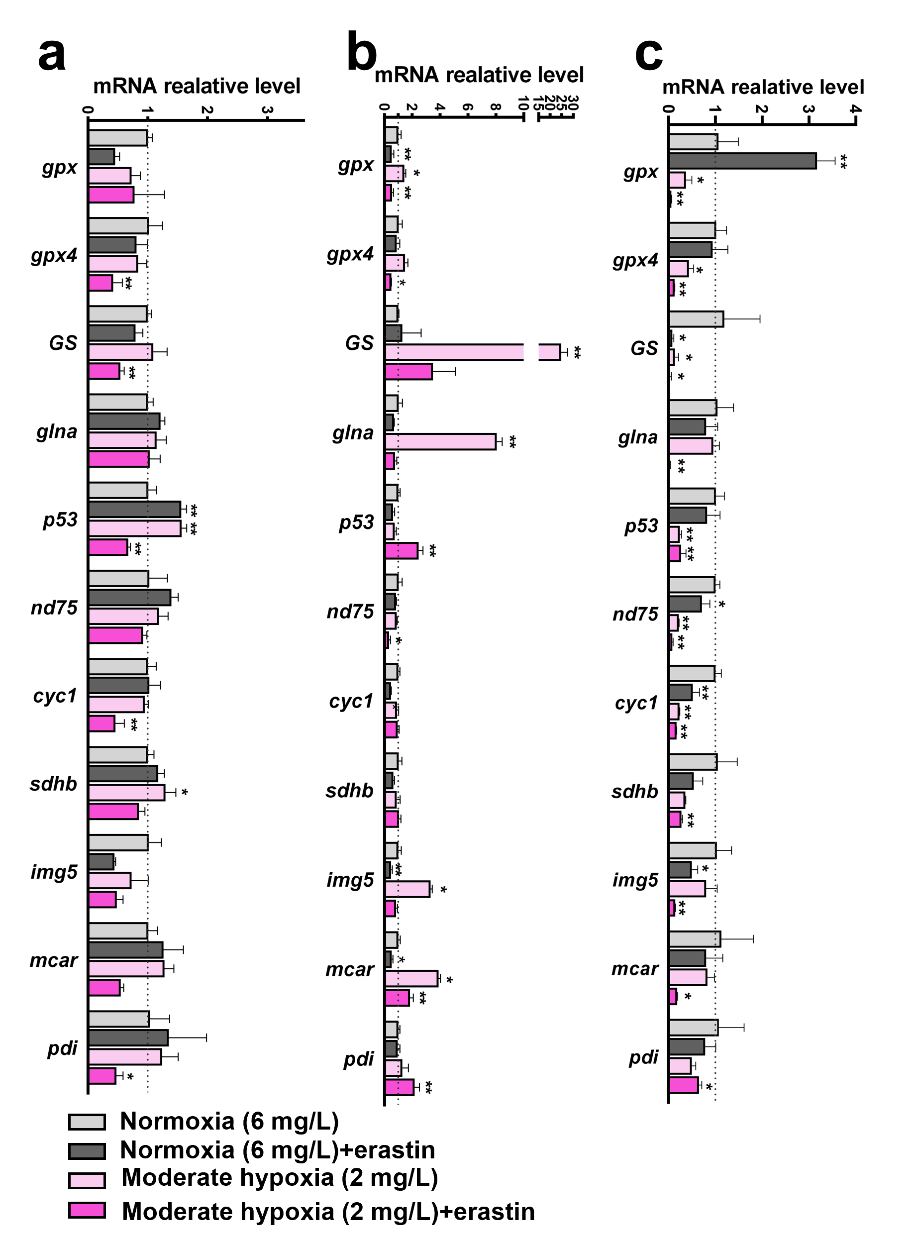


**Supplemental Figrue S6**. mRNA expression of ferroptosis-related genes in *Cryptocaryon irritans* tomonts treated with erastin under normoxia and hypoxia. (a) 6, (b) 24, and (c) 48 h post-treatment (n=3). Statistical analysis was performed using one-way ANOVA with Dunnett's multiple comparisons test. Data are shown as mean ± SD. ‘n’ indicates biological replicates. **p<0.05* and ***p<0.01*.
